# Primary Cutaneous Anaplastic Large Cell Lymphoma—A Review of Clinical, Morphological, Immunohistochemical, and Molecular Features

**DOI:** 10.3390/cancers15164098

**Published:** 2023-08-14

**Authors:** Carlos Ortiz-Hidalgo, Sergio Pina-Oviedo

**Affiliations:** 1 Department of Anatomic Pathology, Fundación Clínica Médica Sur, Mexico City 14050, Mexico; 2 Department of Tissue & Cell Biology, Universidad Panamericana School of Medicine, Mexico City 03920, Mexico; 3 Department of Pathology, Duke University Medical Center, Durham, NC 27710, USA; sergio.pinaoviedo@duke.edu

**Keywords:** primary cutaneous anaplastic large cell lymphoma, CD30, cutaneous T-cell lymphomas, ALK, immunohistochemistry, DUSP22

## Abstract

**Simple Summary:**

Primary cutaneous anaplastic large cell lymphoma (ALCL) is the second most common cutaneous T-cell lymphoma after mycosis fungoides. Although it usually presents as a localized nodule or papule (>2 cm), multifocal lesions may occur in some cases. Patients have an overall good prognosis either in localized or multifocal disease. Microscopically, this neoplasm consists of a dermal infiltrate of medium to large anaplastic cells that may extend to the subcutis. By immunohistochemistry, this tumor is strongly positive for CD30. Primary cutaneous ALCL can mimic several reactive skin conditions as well as other lymphoproliferative disorders, such as lymphomatoid papulosis, more aggressive primary cutaneous lymphomas, or systemic lymphomas involving the skin. Therefore, it is crucial to know the clinical presentation before establishing a diagnosis of primary cutaneous ALCL. Here, we review the clinical and histopathological features of primary cutaneous ALCL as well as its differential diagnosis and most common genetic alterations known to date.

**Abstract:**

Primary cutaneous anaplastic large cell lymphoma (ALCL) is the second most common cutaneous T-cell lymphoma after mycosis fungoides and belongs to the spectrum of cutaneous CD30+ T-cell lymphoproliferative disorders. Although primary cutaneous ALCL usually presents as a localized nodule or papule with or without ulceration, multifocal lesions may occur in up to 20% of cases. Histologically, primary cutaneous ALCL consists of a diffuse dermal infiltrate of medium to large anaplastic/pleomorphic cells with abundant amphophilic-to-eosinophilic cytoplasm, horseshoe-shaped nuclei, strong and diffuse expression of CD30, and with focal or no epidermotropism. The neoplastic infiltrate may show angiocentric distribution and may extend to the subcutis. Patients with localized or multifocal disease have a similar prognosis with a 10-year overall survival rate of 90%. Approximately 30% of primary cutaneous ALCLs harbor a *DUSP22* (6p25.3) gene rearrangement that results in decreased expression of this dual-specific phosphatase, decreased STAT3 activation, and decreased activity of immune and autoimmune-mediated mechanisms regulated by T-cells.

## 1. Introduction

Cutaneous T-cell lymphomas represent a heterogeneous group of non-Hodgkin lymphomas that involve the skin and account for approximately 65% to 75% of all cutaneous lymphomas [1,2,3]. This group includes the CD30+ T-cell lymphoproliferative disorders which, according to the 2022 5th edition of the World Health Organization (WHO) and the European Organization for Research and Treatment of Cancer (EORTC), include lymphomatoid papulosis, primary cutaneous anaplastic large cell lymphoma (ALCL), and borderline lesions [3,4,5,6]. All these entities account for 30% of all cutaneous T-cell lymphomas and are the second most common group of cutaneous T-cell lymphomas after mycosis fungoides and Sezary syndrome [5].

Lymphomatoid papulosis and primary cutaneous ALCL represent opposite extremes in the spectrum of the same disease. Both are composed of an infiltrate of medium to large atypical CD30+ neoplastic T-cells; however, their clinical presentation, evolution, and outcome are different [7,8]. Therefore, a diagnosis of lymphomatoid papulosis or primary cutaneous ALCL should only be established after an appropriate correlation with the clinical presentation and staging [8].

One of the first descriptions of primary cutaneous ALCL was presented by Berti et al. who, in 1989, described the case of a 74-year-old woman with an ulcerated cutaneous nodule in the gluteal region composed of neoplastic epithelioid cells, which were initially interpreted as a metastasis, but were positive by immunohistochemistry for CD45R0 (UCHL-1) and Ber-H2 (CD30). At the time of diagnosis, the patient had no evidence of systemic lymphoma, hence the name “primary anaplastic large cell lymphoma of the skin” given by the authors [9]. Additionally, emphasis was placed on the favorable course of the lesion without the need of chemotherapy [9].

Here, we present a review of the clinical, histological, and immunohistochemical characteristics of primary cutaneous ALCL, including information related to differential diagnosis, as well as the most recent genetic and molecular findings identified in this cutaneous CD30+ T-cell lymphoma.

## 2. Clinical Presentation

Primary cutaneous ALCL comprises about 9% of all cutaneous T-cell lymphomas. It is more common in men (mean age 60 years) and affects Caucasians more frequently [10]. It has rarely been described in children and, to the best of our knowledge, there is a single congenital case documented to date [11,12]. By definition, no systemic ALCL should be identified at the time of diagnosis or 6 months thereafter [13].

**Figure 1:**
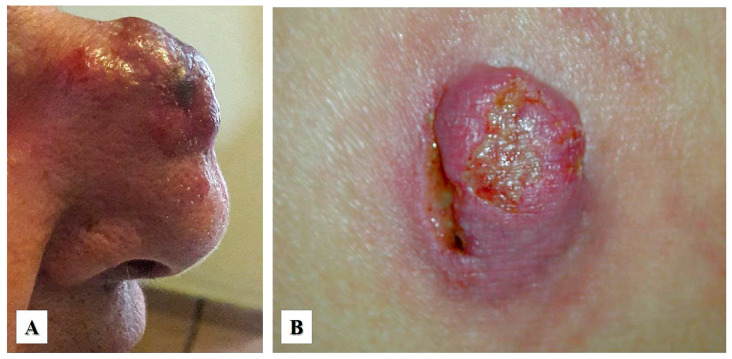
Clinical features of primary cutaneous anaplastic large cell lymphoma. (**A**) A 54-year-old woman with a single rapid-growing lesion on the nasal dorsum. The histologic diagnosis was primary cutaneous anaplastic large cell lymphoma (Courtesy of Dr. Sonia Toussaint-Caire, Mexico City). (**B**) A 35-year-old woman with a single, rapid-growing ulcerated nodular lesion located in the right buttock. The histologic diagnosis was “neutrophil-rich” variant of primary cutaneous anaplastic large cell lymphoma (Courtesy of Dr. Clemente Moreno-Collado, Mexico City).

On clinical exam, primary cutaneous ALCL presents as a red-brown papule or nodule (usually larger than 2 cm), either as an isolated lesion or in clusters, with a rapid growth and common ulceration typically located in the face, trunk, and/or extremities (Figure 1A,B). In contrast to lymphomatoid papulosis, primary cutaneous ALCL does not typically undergo spontaneous regression, which in ALCL has been documented in less than 20% of cases [6,13]. Local recurrence is common but progression to systemic disease is rare [14]. Extracutaneous disease occurs in about 10% of cases, usually involving regional lymph nodes. In general, primary cutaneous ALCL has a favorable prognosis with a 10-year overall survival rate of 90% [15]. However, studies have shown that patients with lesions located in the upper and lower extremities have a worse prognosis [16,17]. Primary cutaneous ALCL can affect immunosuppressed individuals, including those with human immunodeficiency virus (HIV) infection, status post organ transplantation, or those treated with immunomodulatory drugs, such as adalimumab (tumor necrosis factor inhibitor), which is used to treat patients with autoimmune disorders, or fingolimod, which is used to treat patients with relapsing–remitting multiple sclerosis [17,18,19].

Treatment of primary cutaneous ALCL as a single lesion includes complete surgical resection and/or radiation, with most patients achieving complete remission. For patients with local lymph node involvement, radiation of the primary lesion and the local lymph nodes has been recommended, while addition of radiotherapy to surgical excision does not appear to add benefit to localized disease [20]. Relapsed localized disease may be treated as described above, while multifocal and/or refractory disease may benefit from additional chemotherapy, ranging from topical or intralesional agents (imiquimod, interferon-alpha, bexarotene) to systemic therapy using methotrexate or brentuximab-vedotin, or single agent therapy with romidepsin, pralatrexate, gemcitabine, etoposide, or liposomal doxorubicin [20,21,22,23].

## 3. Histopathology

**Figure 2:**
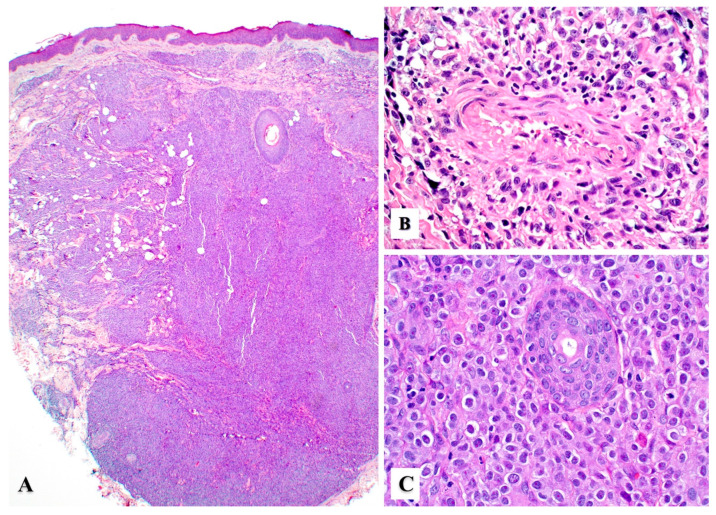
Histopathologic features of primary cutaneous anaplastic large cell lymphoma. (**A**) Sheets of large lymphoma cells with extensive dermal and subcutaneous tissue involvement with no epidermotropism. (**B**) Angiocentric distribution may be seen in some cases and mimic other angiotropic lymphomas (see text). (**C**) Sheets of large atypical lymphoid cells with prominent nucleoli replacing the dermis. An eccrine duct (center) is seen surrounded by tumor cells.

**Figure 3:**
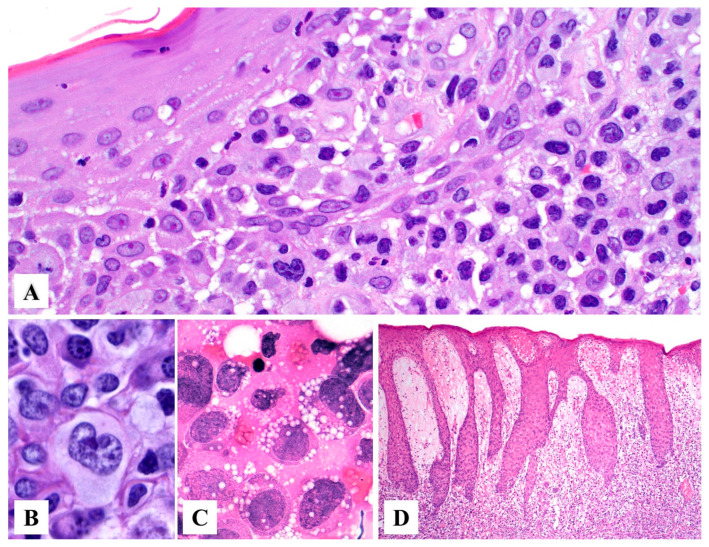
Histopathologic features of primary cutaneous anaplastic large cell lymphoma. (**A**) The papillary dermis is replaced by sheets of large pleomorphic atypical cells with abundant amphophilic to basophilic cytoplasm and kidney-shaped nuclei (“hallmark” cells). (**B**) Higher magnification of a “hallmark” cell. (**C**) The lymphoma cells may occasionally contain abundant cytoplasmic vacuoles. (**D**) Pseudoepitheliomatous hyperplasia in a case of primary cutaneous anaplastic large cell lymphoma.

Primary cutaneous ALCL consists of a diffuse dermal infiltrate of medium to large anaplastic, pleomorphic, or immunoblastic cells with abundant cytoplasm with focal or no epidermotropism [15,24]. The neoplastic infiltrate may show angiocentric distribution and may extend to the subcutis (Figure 2A–C). In 1998, Benharroch et al. described so-called “hallmark” cells in ALCL, which are large (15–50 μm) pleomorphic cells with eccentric “horseshoe” or “kidney-shaped” nuclei, often containing multiple small basophilic nucleoli and abundant amphophilic/basophilic cytoplasm with a prominent eosinophilic Golgi region [25]. These “hallmark” cells are also present in primary cutaneous ALCL (Figure 3A,B) [26]. Occasionally, the neoplastic cells may feature nuclear pseudo-inclusions (so-called “doughnut” cells) [26] or show Reed–Sternberg-like morphology. The tumor cells may also contain numerous cytoplasmic vacuoles (Figure 3C). Up to 20% of cases may not exhibit significant anaplasia, but this finding does not have any prognostic implications [2]. Pseudoepitheliomatous hyperplasia can occur in 20–30% of cases (Figure 3D) [27].

### Morphologic Variants of Primary Cutaneous ALCL

**Figure 4:**
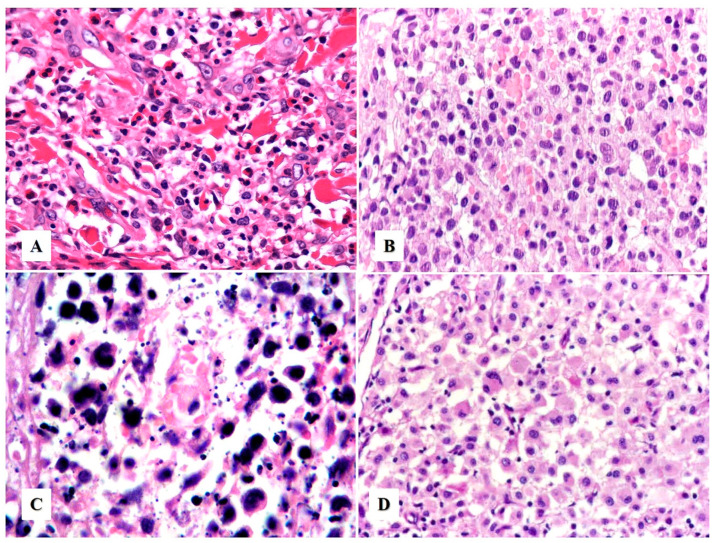
Histologic variants of primary cutaneous anaplastic large cell lymphoma. (**A**) The “neutrophil/eosinophil-rich variant” of anaplastic large cell lymphoma, also known as pyogenic cutaneous lymphoma, may mimic a reactive inflammatory or infectious process (see text). (**B**) The “small cell variant” is extremely challenging to initially recognize as an anaplastic large cell lymphoma. (**C**) Primary cutaneous anaplastic large cell lymphoma with numerous apoptotic bodies can resemble extranodal NK/T-cell lymphoma or cutaneous gamma-delta T-cell lymphoma. (**D**) “Lymphohistiocytic variant” of anaplastic large cell lymphoma may be confused with a histiocyte-rich inflammatory process or an infection by an atypical mycobacteria.

*Neutrophil/eosinophil-rich variant.* This variant is also known as “pyogenic cutaneous lymphoma” and is commonly seen in immunosuppressed individuals [28,29]. It is composed of a robust inflammatory infiltrate of neutrophils, eosinophils, and reactive small T-cells that accompany the large neoplastic cells, creating a challenging distinction from certain subtypes of lymphomatoid papulosis. This variant typically presents as ulcers with numerous neutrophils and intratumoral abscesses (Figure 1B and Figure 4A) and may be associated with pseudoepitheliomatous hyperplasia (Figure 3D). Neutrophils are attracted to the tumor via interleukin-8 (IL-8) produced by the ALCL cells, and an elevated IL-8 can be detected in the serum of these patients [28,29,30].

*Angiocentric/angiodestructive variant.* This variant of primary cutaneous ALCL was described by Kempf et al. in 2013 and resembles the angioinvasive variant of lymphomatoid papulosis (so-called lymphomatoid papulosis type E) [31]. It cannot be entirely excluded that this variant may precede “angiocentric” lymphomatoid papulosis, further supporting the concept that lymphomatoid papulosis and primary cutaneous ALCL are different ends of the spectrum of the same clinicopathological entity [31]. The differential diagnosis of this variant also includes several other cutaneous angiocentric lymphomas (see Section 5).

*“Epidermotropic” variant (DUPS22-rearranged).* In 2019, Onaindia et al. reported cases of primary cutaneous ALCL harboring a translocation involving the *DUSP22-IRF4* gene (located at 6p25.3) [32]. This variant is characterized by a biphasic histopathological pattern composed of (1) transformed CD30+ medium to large lymphocytes with abundant finely granular cytoplasm, numerous mitoses, and apoptosis with diffuse infiltration into the dermis; and (2) smaller atypical CD30+ lymphocytes infiltrating the epidermis in a pattern resembling Woringer–Kolopp disease (pagetoid reticulosis) with occasional “Pautrier-Darier-like” micro-abscesses [32,33].

*Other less common variants.* These include cases with (1) small cell morphology (Figure 4B), (2) numerous apoptotic bodies (Figure 4C), (3) lymphohistiocytic morphology (Figure 4D), (4) lymphovascular invasion, [34] (5) intravascular localization [35], (6) keratoacanthomatous hyperplasia [36], (7) myxoid stroma, or (8) spindle cell/sarcomatoid morphology [37,38]. None of these variants appear to have better or worse prognostic value in primary cutaneous ALCL.

## 4. Immunohistochemistry

**Figure 5:**
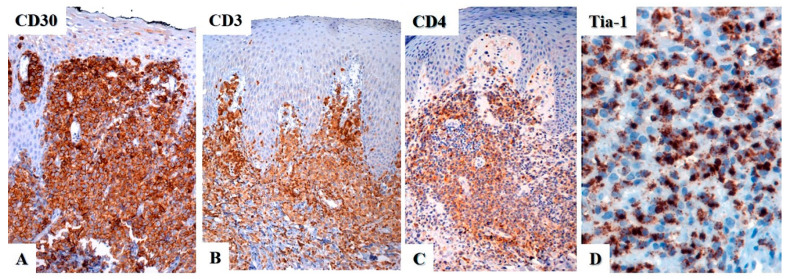
Immunohistochemistry in primary cutaneous anaplastic large cell lymphoma. (**A**) CD30 is strongly and diffusely positive in the neoplastic cells with a membranous and paranuclear dot (Golgi) pattern. (**B**) CD3 is positive in some cases, whereas in others it may be negative or only weakly positive. (**C**) The tumor cells are usually positive for CD4 and for (**D**) cytotoxic markers, in this case TIA-1.

Primary cutaneous ALCL has an identical immunophenotype to ALK-negative systemic ALCL. By definition, CD30 should be positive in more than 75% of the tumor cells (Figure 5A). Therefore, if the expression of CD30 is weak or partial, it should be considered that the skin involvement is due to another type of T-cell lymphoma and not primary cutaneous ALCL [13]. CD30—also known as Ki-1 or TNFRSF8—is a 120 kDa transmembrane glycoprotein receptor expressed in different types of B- and T-cell lymphomas, in plasma cells, in a subtype of activated macrophages, in myeloblasts in a subset of acute myeloid leukemia, and in some cases of myelodysplastic syndrome [22]. CD30 is also a marker of B-cell and T-cell activation, and is inducible in vitro by mitogenic signaling and viral stimulation; in regard to skin histopathology, it can also be positive in several non-neoplastic skin infiltrates, such as arthropod bites (ticks, scabies), various infections (*Leishmania*, syphilis, herpes simplex virus, varicella zoster virus, *Molluscum contagiosum*), hidradenitis, rhinophyma, certain drug eruptions, and in stasis ulcers [22].

Most cases of primary cutaneous ALCL are positive for CD45, CD43, MUM1/IRF4, and CD4 (Figure 5B,C) [6], with variable expression of CD2, CD5, CD7, and CD45RO. CD3 may be negative or weakly expressed (Figure 5B) due to genetic alterations of the T-cell receptor (TCR) in the tumor cells [39]. A study by Wechsler et al. from 2022 showed that 90% of CD30+ T-cell lymphoproliferative disorders (29 cases of lymphomatoid papulosis, 20 cases of primary cutaneous ALCL) showed loss of at least one T-cell antigen, with loss of CD7 being the most common (86% of cases), followed by loss of CD5 and CD3 (28% and 26% of cases, respectively), but with overall preservation of CD2 expression (loss only in 10% of cases) [40]. Close to 20% of cases are positive for CD8 and about 25% may be double negative for CD4 and CD8 [41]. Primary cutaneous ALCL may show a cytotoxic immunophenotype with expression of perforin, T-cell intracellular antigen-1 (TIA-1), and granzyme B (Figure 5D). CD15 can be positive in up to 40% of cases and CD56 is almost always negative, with one study reporting only 1.4% (2/148) of cases positive for this marker [42]. Epithelial membrane antigen (EMA) is expressed less frequently than in systemic ALCL. Epstein–Barr virus (EBV) is always negative, either by immunohistochemistry using LMP-1 or by in situ hybridization with EBV-encoded RNA (EBER).

ALK (CD246) is negative in primary cutaneous ALCL. However, reports of ALK+ cases limited to the skin have been documented in the pediatric population [43,44,45]. Unlike systemic ALCL, cases of ALK+ primary cutaneous ALCL appear to have a favorable outcome similar to that of patients with ALK-negative primary cutaneous ALCL. There are no specific morphological features that can distinguish ALK+ from ALK-negative cases.

GATA3, a zinc finger transcription factor involved in the control of CD4+ effector T-cell differentiation, is negative or weakly positive in primary cutaneous ALCL [46]. Mitteldorf et al. showed that galectin 3 (Gal-3), a β-galactoside binding protein, is expressed in primary cutaneous ALCL [47]. Clusterin, a ubiquitous 80 kDa heterodimeric glycoprotein, is also positive in primary cutaneous ALCL with a paranuclear dot/Golgi pattern. Even though clusterin is also positive in other hematological malignancies, its characteristic labeling pattern in entities other than primary cutaneous ALCL is diffuse cytoplasmic and/or membranous [48]. The expression of CD71 (transferrin receptor-1), HLA-DR, and CD25 (IL-2 receptor alpha chain) has been reported in approximately half of cases [17]. Similarly, about 50% of cases express CLA (cutaneous lymphocyte antigen), which it is usually negative in systemic ALCL that has not involved the skin [49].

## 5. Differential Diagnosis

The differential diagnosis of primary cutaneous ALCL includes a wide variety of primary cutaneous and systemic lymphomas with skin involvement composed of large lymphocytes [6,15,24,39]. As previously mentioned, primary cutaneous ALCL belongs to the group of CD30+ T-cell lymphoproliferative disorders, and the distinction between other subtypes within this group requires the integration of clinical, histological, and immunohistochemical findings, and occasionally the results from genetic and/or molecular alterations. See also Table 1.

### 5.1. Differential Diagnosis with Other CD30+ T-Cell Lymphoproliferative Disorders

*Systemic ALCL involving skin*. One the most important differential diagnoses to consider is that of primary cutaneous ALCL versus systemic ALCL with secondary skin involvement. In this instance, ALK immunohistochemistry is useful to differentiate between these two entities. About 50% of cases of systemic ALCL harbor the t(2;5) (p23;q35) (*ALK-NMP1*) or other *ALK* rearrangements, whereas this genetic alteration has only rarely been reported in primary cutaneous ALCL [41]. However, this is not the case for systemic ALK-negative ALCL with skin involvement. For these cases, the clinical presentation and prior history are mandatory to support or exclude secondary cutaneous involvement by lymphoma or primary cutaneous ALCL. In addition, strong and diffuse positivity for EMA suggests secondary skin involvement by systemic ALCL rather than primary cutaneous ALCL [31].

*Lymphomatoid papulosis types C and E*. Because there is a significant histological overlap between lymphomatoid papulosis diffuse large cell type (so-called lymphomatoid papulosis type C) and primary cutaneous ALCL, it is extremely challenging—if not impossible—to separate these lesions by morphology. The presence of clusters of red or purple papules and small nodules on the extremities (usually smaller than 2 cm) at different stages of development that show spontaneous regression in a few weeks is characteristic of lymphomatoid papulosis and excludes primary cutaneous ALCL. The “angiocentric” variant of primary cutaneous ALCL is difficult to distinguish from the “angioinvasive” subtype of lymphomatoid papulosis (so-called lymphomatoid papulosis type E). As with other lymphomatoid papulosis subtypes, their distinction can only be established by clinical correlation. Importantly, it cannot be entirely excluded that the “angiocentric” primary cutaneous ALCL may precede “angiocentric” lymphomatoid papulosis, further supporting the concept that these two disorders represent different ends of the spectrum of the same clinicopathological entity [31].

*Mycosis fungoides with CD30+ large cell transformation.* In these cases, clinical history is mandatory. A prior history of mycosis fungoides rules out a diagnosis of primary cutaneous ALCL. However, clinical information may not be available at the time of pathologic review, and unrecognized cases of mycosis fungoides with large cell transformation may rarely present initially as a large cutaneous mass. In addition to prior clinical history, the expression of GATA3 and galectin-3 appears to be helpful. GATA3 is strong and diffuse in mycosis fungoides with CD30+ large cell transformation but negative or only weakly positive in primary cutaneous ALCL [46]. On the other hand, galectin-3 is positive in primary cutaneous ALCL but has a lower expression in mycosis fungoides with large cell transformation [47]. Unfortunately, galectin-3 is not a routine antibody available in most pathology laboratories.

### 5.2. Differential Diagnosis with Entities Other Than CD30+ T-Cell Lymphoproliferative Disorders

*Reactive/inflammatory cutaneous conditions.* The “neutrophil/eosinophil-rich” variant of primary cutaneous ALCL (see above) can be confused with various inflammatory diseases such as pyoderma gangrenosum, Sweet syndrome, facial pyoderma, and deep fungal infections [28,29]. In this instance, a strong and diffuse expression of CD30 on the atypical lymphoid cells confirms primary cutaneous ALCL and rules out any of the aforementioned inflammatory disorders, which only contain a few scattered CD30+ immunoblasts.

*Angiocentric lymphomas involving skin.* Given the angiodestructive pattern seen in “angiocentric” primary cutaneous ALCL, the differential diagnosis includes extranodal/cutaneous NK/T-cell lymphoma, adult T-cell leukemia/lymphoma, cutaneous γ/δ T-cell lymphoma, and hydroa vacciniforme-like lymphoproliferative disorder [31]. Most of these can be excluded by features in the clinical presentation, somewhat different morphology (usually with small to intermediate-sized lymphoma cells), different immunophenotype along with lack of CD30 expression, positivity for EBV (NK/T-cell lymphoma and hydroa vacciniforme-like lymphoproliferative disorder), or positivity for HTLV-1 (adult T-cell leukemia/lymphoma).

*Classic Hodgkin lymphoma involving skin.* As mentioned previously, CD15 can be positive in up to 40% of primary cutaneous ALCL cases, posing a diagnostic difficulty with classic Hodgkin lymphoma. However, it should be noted that cutaneous classic Hodgkin lymphoma is exceedingly rare, and it is usually seen in patients with known widespread disease. In fact, a case of “classic Hodgkin lymphoma” involving skin should alert the pathologist about a lymphoproliferative disorder secondary to immunosuppression or iatrogenic lymphoproliferative disorder involving skin. On morphology, the distinction is obvious, with primary cutaneous ALCL showing sheets of large pleomorphic cells, “hallmark” cells, and occasional Reed–Sternberg-like cells. These morphologic features are not seen in classic Hodgkin lymphoma. Moreover, unlike classic Hodgkin lymphoma, primary cutaneous ALCL rarely expresses PAX5 and is negative for EBV (which may be positive or negative in classic Hodgkin lymphoma) [6,50].

*B-cell lymphoma with plasmablastic differentiation involving skin.* The morphologic distinction may be difficult; however, the immunophenotype readily solves this issue since this is a B-cell lymphoma despite the expression of CD30. Additionally, these lymphomas are usually positive for EBV, excluding primary cutaneous ALCL [13].

## 6. Genetic and Molecular Features

The pathogenetic mechanisms of CD30+ T-cell lymphoproliferative disorders are not entirely known. Interestingly, the presence of a rearrangement of the dual-specific protein phosphatase 22 (*DUSP22*) gene located at 6p25.3 in cases of lymphomatoid papulosis and in primary cutaneous ALCL supports the concept that both entities have a related pathogenesis. Close to 30% of primary cutaneous ALCL cases harbor a *DUSP22* rearrangement, making this the most common genetic abnormality in this neoplasm known to date [51,52,53]. The *DUSP22* gene product is an enzyme of the same name (DUSP22), which belongs to the subfamily of specific dual phosphatases that regulate mitogen-activated protein kinases (MAPKs) associated with proliferation and cell differentiation [7,54]. The *DUSP22* rearrangement decreases expression of the DUSP22 protein, which decreases the activation of STAT3 (Signal transducer and activator of transcription 3) induced by IL-6. DUSP22 also participates in the inactivation of the LCK (lymphocyte-specific protein tyrosine kinase) pathway, resulting in reduced immunity and autoimmunity mediated by T-cells [54]. Therefore, it is possible that DUSP22 plays an important role in inflammatory processes and in various T-cell lymphoproliferative disorders [55].

Comparative genomic hybridization studies conducted in primary cutaneous ALCL have identified chromosomal instability in up to 40% of cases in *FGFR1* (8p11), *NRAS* (1p13.2), *MYCN* (2p24.1), *RAF1* (3p25), *CTSB* (8p22), *FES* (15q26.1), and *CBFA2* (21q22.3) genes [56]. Likewise, gains in chromosomes 7q31 and 17q and losses in regions 3p, 6q16–6q21, 6q27, and 13q34 have also been detected in this cutaneous lymphoma [56,57]. The clinical significance of these findings has not yet been established and chromosomal alterations that can distinguish between lymphomatoid papulosis and primary cutaneous ALCL have yet to be identified. In 2014, using whole-transcriptome sequencing, Velusamy et al. identified a chimeric fusion involving *NPM1* (5q35) and *TYK2* (19p13) that encodes an NPM1-TYK2 protein in 12.5% (4/32) of cases of primary cutaneous ALCL and 20% (3/15) of cases of lymphomatoid papulosis [58]. This fusion protein induces STAT signaling and represents a therapeutic target in this specific group of CD30+ T-cell lymphoproliferative disorders [58].

## 7. Conclusions

Primary cutaneous ALCL is a CD30+ T-cell lymphoproliferative disorder having a relatively favorable prognosis, with a 10-year survival rate of 90% in the absence of advanced stage disease. The diagnosis of primary cutaneous ALCL is largely based on clinicopathologic correlation given the morphologic, immunophenotypic, and molecular overlap with some subtypes of lymphomatoid papulosis and with some other more aggressive hematolymphoid neoplasms primary or secondarily involving the skin. Treatment includes surgical resection, local radiotherapy, cyclophosphamide/doxorubicin/vincristine/prednisolone (CHOP) chemotherapy, or a combination of these modalities [50], depending on the severity of the disease. CD30 is not only an important diagnostic and prognostic marker, but also a therapeutic target for antibody-based therapy, such as brentuximab-vedotin, which is approved for relapsed or refractory cutaneous CD30+ T-cell lymphomas, including primary cutaneous ALCL [17,20,39,59,60]. Further discoveries await to better characterize the pathogenesis of this disorder.

## Figures and Tables

**Table 1:** Differential diagnosis of primary cutaneous anaplastic large cell lymphoma.

**Lymphomatoid papulosis, type C (diffuse large cell type)** Papular, papulonecrotic, or nodular lesions (usually smaller than 1 cm) Lesions resolve spontaneously (some authors consider this variant a borderline lesion with primary cutaneous anaplastic large cell lymphoma) Groups of large CD30+ T-cells with few admixed background inflammatory cells Morphologically indistinguishable from primary cutaneous anaplastic large cell lymphoma, except for minimal subcutaneous involvement (a “soft” finding to favor lymphomatoid papulosis)
**ALK+ systemic anaplastic large cell lymphoma with skin involvement** Skin involvement occurs in up to 60% of cases Usually affects the pediatric population or young adults Involvement of lymph nodes and extranodal sites Strong and diffuse expression of CD30, ALK, and EMA *ALK* (2p23) gene rearrangement present Rare cases of primary cutaneous anaplastic large cell lymphoma in children can be ALK+
**ALK-negative systemic anaplastic large cell lymphoma with skin involvement** Skin involvement may occur in up to 20% of cases Older adults Involvement of lymph nodes and extranodal sites Morphologically and immunophenotypically indistinguishable from primary cutaneous anaplastic large cell lymphoma, distinction can only be made based of clinical presentation
**Mycosis fungoides with CD30+ large cell transformation** It occurs in 20% of advanced cases of mycosis fungoides Clinical picture of mycosis fungoides Histological features of mycosis fungoides may or may not be present CD3+, CD5(−) and/or CD7(−) or reduced expression of these markers Variable CD30+ GATA3+ Bad prognosis
**Classic Hodgkin lymphoma involving skin** Very rare as initial presentation (0.5–3.5% of cases) Consider first a “Hodgkin-like” lymphoproliferative disorder arising in the setting of immunosuppression or an iatrogenic lymphoproliferative disorder (history of immunomodulatory therapy, etc.) CD30+, CD15+/−, frequently Epstein–Barr virus positive PAX5+ with weak nuclear labeling CD45(−), T-cell markers (−)
**Peripheral T lymphoma, not otherwise specified, involving skin** Common B symptoms Systemic and lymph node involvement Usually, no prior skin condition Anaplastic cells and “hallmark” cells may or may not be present CD30(−), weak or focal (less than 75% of tumor cells)
**Subcutaneous panniculitis-like T-cell lymphoma** More common in women, median age 35 years 20% of patients with associated autoimmune disease, most commonly systemic lupus erythematosus Lymphoid infiltrate confined to subcutaneous tissue (lobular involvement) CD8+, cytotoxic markers (TIA-1, granzyme B, perforin)+ CD30(−), CD56(−), CD123(−) TCRαβ+
**Primary cutaneous gamma-delta T-cell lymphoma** Aggressive clinical presentation (median survival 12 months) Generalized skin disease; predominantly affects the extremities Dissemination to mucous membranes is frequent Common B symptoms, accompanied by hemophagocytic syndrome Three histologic patterns: (1) epidermotropic, (2) dermal and (3) subcutaneous Infiltrate of medium/large lymphocytes with variable degrees of apoptosis, necrosis and angioinvasion CD2+, CD3+, CD7+/−, CD56+, cytotoxic markers (TIA-1, granzyme B, perforin)+ CD30/+ (usually less than 75% of tumor cells) Most cases CD5(−), double negative CD4(−)/CD8(−) TCRγ/δ+
**Primary cutaneous CD8+ aggressive epidermotropic cytotoxic T-cell lymphoma** Aggressive clinical presentation (median survival 12 months) Skin lesions, nodules, and ulcerated plaques May spread to visceral organs (lung, testis, central nervous system) Epidermotropic, pagetoid distribution with ulceration and necrosis Common folliculotropism and syringotropism It may be morphologically indistinguishable from lymphomatoid papulosis type D CD3+, CD8+, variable CD7, CD2(−), cytotoxic markers (TIA-1, granzyme B, perforin)+, high Ki-67 CD30−/+ (usually less than 75% of tumor cells) TCRαβ+
**Primary cutaneous CD4+ small/medium T-cell lymphoproliferative disorder** Solitary plaques or nodules affecting the face, neck, and upper trunk Excellent prognosis Small/medium-sized lymphocytes, mildly pleomorphic, less than 30% are large lymphocytes Mixture of plasma cells, macrophages, and occasional multinucleated giant cells CD3+, CD4+, PD-L1+, bcl-6+, CXCL13+, low Ki-67 (less than 20%) CD8(−), cytotoxic markers (TIA-1, granzyme B, perforin)(−) CD30(−)

## Data Availability

Not applicable.
